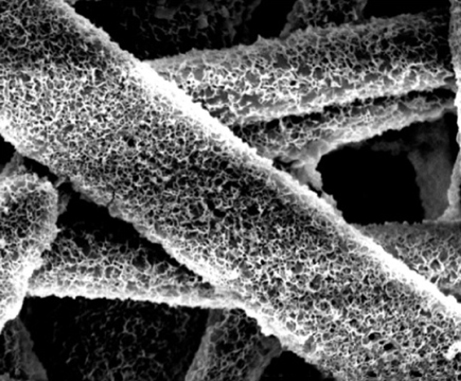# A humanised xenograft mouse model for studying bone metastasis

**Published:** 2014-02

Bone is a common site for metastases formation, particularly tumours that originate in breast tissue. Early-stage detection of bone metastases is challenging and, once established, the disease is virtually incurable. This highlights the urgent need to develop new approaches for clinical management of the disease. Unfortunately, efforts towards this have been hampered by the lack of suitable animal models that recapitulate human bone metastasis. To address this challenge, Dietmar W. Hutmacher and colleagues have now developed a humanised xenograft mouse model of breast-cancer-induced bone metastasis. They seeded biodegradable composite scaffolds with human bone-forming cells and implanted the construct together with an osteoinductive growth factor, creating a viable ‘organ’ bone in mice. After injection with human breast cancer cells, metastases were detected in the humanised bone. Thus, the study establishes a unique *in vivo* model that mimics human breast cancer metastasis to a human-bone-like environment, providing a tool for exploring bone metastasis and evaluating potential therapies. Page 299

**Figure:**